# Supplementary material for: Target cell-specific synaptic dynamics of excitatory to inhibitory neuron connections in supragranular layers of human neocortex
Source: eLife. 2023 May 30;12:e81863. doi: 10.7554/eLife.81863 (PMC10332811; doi:10.7554/eLife.81863)
Supplement: Supplementary file 1. [file elife-81863-supp1.docx]

**Supplementary File 1. PVALB versus SST subclass differential gene expression analysis.**

p_val and p_val_adj indicate significance and adjusted significance of the differential expression test (Wilcoxon sum rank test). avg_log2FC means the average log2 fold change in expression between the two cell populations (PVALB and SST). pct.1 is the proportion of target nuclei a gene is expressed in, and pct.2 the proportion of the background population the gene is expressed in.

**1) List of conserved PVALB markers**

|  | genes | Human  _p_val | Human_avg  _log2FC | Human  _pct.1 | Human  _pct.2 | Human_p  _val_adj | Mouse  _p_val | Mouse_avg  _log2FC | Mouse  _pct.1 | Mouse  _pct.2 | Mouse_p  _val_adj |
| --- | --- | --- | --- | --- | --- | --- | --- | --- | --- | --- | --- |
| 1 | ***ERBB4*** | 5.16E-247 | 3.909283 | 1 | 0.946 | 7.67E-243 | 0 | 2.702707 | 1 | 0.802 | 0 |
| 2 | ***GRIA4*** | 4.15E-226 | 2.736456 | 1 | 0.805 | 6.18E-222 | 3.13E-131 | 0.702254 | 0.999 | 0.994 | 4.65E-127 |
| 3 | ***SHISA9*** | 1.34E-222 | 2.598381 | 0.997 | 0.909 | 2.00E-218 | 9.98E-287 | 0.936583 | 0.928 | 0.315 | 1.48E-282 |
| 4 | ***ZNF804A*** | 3.74E-199 | 2.712085 | 0.995 | 0.847 | 5.56E-195 | 2.25E-244 | 1.087752 | 0.963 | 0.6 | 3.35E-240 |
| 5 | ***FGF12*** | 3.26E-189 | 1.218129 | 1 | 1 | 4.85E-185 | 2.57E-247 | 0.90732 | 1 | 1 | 3.82E-243 |
| 6 | ***KCNC2*** | 8.03E-175 | 1.471195 | 1 | 0.987 | 1.19E-170 | 5.60E-211 | 0.946169 | 1 | 1 | 8.32E-207 |
| 7 | ***BTBD11*** | 2.13E-169 | 1.811156 | 0.996 | 0.814 | 3.17E-165 | 3.93E-255 | 0.972218 | 0.982 | 0.856 | 5.84E-251 |
| 8 | ***PPARGC1A*** | 6.64E-168 | 1.483409 | 1 | 0.987 | 9.88E-164 | 3.38E-258 | 1.212143 | 1 | 0.971 | 5.02E-254 |
| 9 | ***GABRA1*** | 1.85E-164 | 1.844888 | 0.993 | 0.641 | 2.75E-160 | 0 | 1.663122 | 0.997 | 0.848 | 0 |
| 10 | ***GABRB2*** | 1.11E-162 | 1.519482 | 1 | 0.912 | 1.65E-158 | 6.35E-213 | 0.920699 | 0.999 | 0.86 | 9.44E-209 |
| 11 | ***SLC4A4*** | 1.40E-161 | 1.62864 | 0.932 | 0.582 | 2.07E-157 | 0 | 0.700453 | 0.924 | 0.269 | 0 |
| 12 | ***FAM19A2*** | 1.64E-145 | 1.729735 | 0.999 | 0.906 | 2.44E-141 | 0 | 1.501664 | 0.978 | 0.427 | 0 |
| 13 | ***PLCXD3*** | 1.63E-144 | 1.806278 | 0.894 | 0.518 | 2.43E-140 | 2.05E-228 | 0.919008 | 0.977 | 0.805 | 3.06E-224 |
| 14 | ***IL1RAPL1*** | 8.55E-143 | 1.182886 | 1 | 1 | 1.27E-138 | 2.80E-282 | 1.351967 | 0.999 | 0.986 | 4.17E-278 |
| 15 | ***CALN1*** | 1.32E-141 | 1.66584 | 0.995 | 0.976 | 1.97E-137 | 2.00E-124 | 0.722693 | 0.616 | 0.274 | 2.97E-120 |
| 16 | ***ZNF536*** | 1.06E-138 | 1.348962 | 0.993 | 0.857 | 1.57E-134 | 5.80E-275 | 0.859355 | 0.994 | 0.59 | 8.63E-271 |
| 17 | ***PLXNA4*** | 6.32E-136 | 1.468606 | 0.992 | 0.766 | 9.40E-132 | 8.99E-268 | 0.923645 | 0.997 | 0.819 | 1.34E-263 |
| 18 | ***SLIT2*** | 2.89E-133 | 1.628283 | 0.999 | 0.778 | 4.30E-129 | 0 | 1.472302 | 0.968 | 0.389 | 0 |
| 19 | ***FMN1*** | 2.42E-130 | 1.328625 | 0.996 | 0.895 | 3.60E-126 | 5.00E-126 | 0.560664 | 0.941 | 0.749 | 7.44E-122 |
| 20 | ***KCNAB1*** | 1.24E-124 | 1.224888 | 0.997 | 0.961 | 1.85E-120 | 1.47E-69 | 0.583964 | 0.965 | 0.895 | 2.18E-65 |
| 21 | ***LRRC4C*** | 5.53E-123 | 1.277739 | 0.996 | 0.964 | 8.22E-119 | 0 | 2.0183 | 0.999 | 0.936 | 0 |
| 22 | ***NDST3*** | 1.48E-122 | 1.514933 | 0.972 | 0.712 | 2.20E-118 | 1.27E-140 | 0.77216 | 0.738 | 0.366 | 1.90E-136 |
| 23 | ***KCNIP2*** | 8.33E-118 | 0.859746 | 0.877 | 0.37 | 1.24E-113 | 8.35E-295 | 1.235439 | 0.958 | 0.522 | 1.24E-290 |
| 24 | ***PVALB*** | 9.33E-115 | 1.006397 | 0.604 | 0.085 | 1.39E-110 | 0 | 3.172998 | 0.971 | 0.396 | 0 |
| 25 | ***GABRD*** | 1.67E-114 | 0.527471 | 0.755 | 0.191 | 2.49E-110 | 0 | 1.430415 | 0.985 | 0.507 | 0 |
| 26 | ***INPP4B*** | 8.35E-114 | 1.334956 | 0.993 | 0.954 | 1.24E-109 | 1.30E-65 | 0.666014 | 0.56 | 0.298 | 1.93E-61 |
| 27 | ***ATRNL1*** | 4.21E-113 | 1.019315 | 1 | 1 | 6.25E-109 | 1.30E-164 | 0.729006 | 0.987 | 0.918 | 1.94E-160 |
| 28 | ***KCNH7*** | 7.70E-111 | 1.232209 | 0.985 | 0.781 | 1.15E-106 | 0 | 1.673473 | 0.999 | 0.686 | 0 |
| 29 | ***IL1RAP*** | 1.99E-106 | 1.021186 | 0.736 | 0.235 | 2.95E-102 | 9.50E-144 | 0.562661 | 0.69 | 0.292 | 1.41E-139 |
| 30 | ***PRKCA*** | 2.10E-105 | 1.182309 | 0.996 | 0.945 | 3.13E-101 | 2.34E-165 | 0.62359 | 0.966 | 0.798 | 3.48E-161 |
| 31 | ***LANCL1*** | 1.93E-103 | 1.228749 | 0.948 | 0.582 | 2.87E-99 | 0 | 0.922588 | 1 | 1 | 0 |
| 32 | ***TAC1*** | 1.49E-101 | 0.825286 | 0.914 | 0.328 | 2.21E-97 | 0 | 2.276749 | 0.845 | 0.168 | 0 |
| 33 | ***VWC2*** | 2.73E-96 | 0.977925 | 0.977 | 0.536 | 4.06E-92 | 1.04E-170 | 0.677306 | 0.951 | 0.615 | 1.54E-166 |
| 34 | ***LRRTM4*** | 3.72E-94 | 1.082301 | 1 | 0.992 | 5.53E-90 | 6.61E-99 | 0.765256 | 0.974 | 0.885 | 9.83E-95 |
| 35 | ***CEMIP*** | 4.59E-93 | 1.243856 | 0.863 | 0.527 | 6.82E-89 | 0 | 1.036275 | 0.893 | 0.255 | 0 |
| 36 | ***SH3RF3*** | 1.47E-91 | 0.840803 | 0.984 | 0.758 | 2.19E-87 | 2.80E-122 | 0.56761 | 0.976 | 0.82 | 4.17E-118 |
| 37 | ***TMEM132C*** | 4.28E-90 | 1.092452 | 0.979 | 0.772 | 6.37E-86 | 1.77E-186 | 0.646135 | 0.936 | 0.572 | 2.63E-182 |
| 38 | ***ESRRG*** | 3.38E-89 | 1.054452 | 0.992 | 0.921 | 5.03E-85 | 0 | 1.17575 | 0.989 | 0.743 | 0 |
| 39 | ***SRRM4*** | 1.84E-88 | 0.972084 | 0.997 | 0.907 | 2.73E-84 | 0 | 0.804017 | 0.996 | 0.554 | 0 |
| 40 | ***GABRG3*** | 2.33E-87 | 1.068223 | 1 | 0.916 | 3.46E-83 | 7.89E-237 | 1.198166 | 0.998 | 0.884 | 1.17E-232 |
| 41 | ***WIF1*** | 3.72E-87 | 0.901688 | 0.797 | 0.33 | 5.54E-83 | 8.08E-198 | 0.703371 | 0.888 | 0.65 | 1.20E-193 |
| 42 | ***KCNAB3*** | 1.73E-84 | 0.670328 | 0.858 | 0.393 | 2.57E-80 | 2.33E-271 | 1.021017 | 0.979 | 0.749 | 3.46E-267 |
| 43 | ***TM6SF1*** | 1.34E-82 | 0.664406 | 0.684 | 0.222 | 1.99E-78 | 6.77E-211 | 0.64646 | 0.984 | 0.754 | 1.01E-206 |
| 44 | ***MEF2C*** | 2.91E-82 | 0.837102 | 1 | 0.989 | 4.33E-78 | 2.97E-278 | 1.184035 | 1 | 0.963 | 4.41E-274 |
| 45 | ***NTRK3*** | 2.96E-82 | 1.002019 | 0.977 | 0.83 | 4.41E-78 | 0 | 1.300106 | 0.99 | 0.609 | 0 |
| 46 | ***TRPC4*** | 1.58E-81 | 0.929117 | 0.967 | 0.691 | 2.35E-77 | 1.60E-184 | 0.68681 | 0.906 | 0.457 | 2.38E-180 |
| 47 | ***OSBPL3*** | 4.63E-80 | 0.890367 | 0.993 | 0.857 | 6.88E-76 | 5.04E-128 | 0.546871 | 0.972 | 0.784 | 7.50E-124 |
| 48 | ***LUZP2*** | 9.37E-75 | 1.869299 | 0.93 | 0.841 | 1.39E-70 | 1.58E-28 | 0.68267 | 0.798 | 0.788 | 2.35E-24 |
| 49 | ***ANKRD29*** | 2.54E-73 | 0.581648 | 0.707 | 0.347 | 3.78E-69 | 1.36E-244 | 0.876352 | 0.981 | 0.784 | 2.02E-240 |
| 50 | ***KCNJ3*** | 2.15E-69 | 0.82566 | 0.993 | 0.896 | 3.19E-65 | 1.70E-175 | 0.742957 | 0.996 | 0.913 | 2.53E-171 |
| 51 | ***TRPS1*** | 1.46E-68 | 0.926962 | 0.893 | 0.605 | 2.17E-64 | 1.08E-157 | 1.008885 | 0.828 | 0.52 | 1.61E-153 |
| 52 | ***TMEM108*** | 3.79E-62 | 0.754134 | 0.997 | 0.971 | 5.63E-58 | 1.20E-238 | 0.851161 | 0.852 | 0.401 | 1.78E-234 |
| 53 | ***IDH3A*** | 1.09E-61 | 0.632919 | 0.845 | 0.529 | 1.62E-57 | 5.57E-266 | 0.653639 | 1 | 0.998 | 8.28E-262 |
| 54 | ***PPFIA2*** | 3.58E-61 | 0.641683 | 1 | 0.996 | 5.33E-57 | 9.44E-97 | 0.582803 | 0.979 | 0.952 | 1.40E-92 |
| 55 | ***MDGA2*** | 5.81E-60 | 0.563017 | 1 | 1 | 8.64E-56 | 5.40E-86 | 0.58719 | 0.999 | 0.995 | 8.03E-82 |
| 56 | ***GPR22*** | 1.23E-59 | 0.760598 | 0.822 | 0.437 | 1.84E-55 | 7.37E-134 | 0.710846 | 0.984 | 0.833 | 1.10E-129 |
| 57 | ***LGI2*** | 7.55E-58 | 0.506303 | 0.598 | 0.203 | 1.12E-53 | 2.95E-236 | 0.720964 | 0.987 | 0.779 | 4.39E-232 |
| 58 | ***SPARCL1*** | 2.56E-55 | 0.770897 | 0.997 | 0.961 | 3.80E-51 | 1.85E-235 | 1.089883 | 1 | 0.982 | 2.75E-231 |
| 59 | ***KCNS3*** | 2.16E-52 | 0.53151 | 0.61 | 0.29 | 3.21E-48 | 1.71E-143 | 0.602152 | 0.948 | 0.613 | 2.54E-139 |
| 60 | ***MDH1*** | 7.20E-52 | 0.857094 | 0.953 | 0.776 | 1.07E-47 | 0 | 0.68976 | 1 | 1 | 0 |
| 61 | ***CNTN5*** | 5.93E-48 | 0.877416 | 1 | 0.999 | 8.82E-44 | 1.80E-120 | 1.574099 | 0.939 | 0.853 | 2.68E-116 |
| 62 | ***ME3*** | 1.72E-41 | 0.521468 | 0.989 | 0.949 | 2.55E-37 | 0 | 1.194677 | 0.993 | 0.881 | 0 |
| 63 | ***ATP5G3*** | 2.10E-41 | 0.619552 | 0.795 | 0.519 | 3.13E-37 | 9.49E-243 | 0.515712 | 1 | 1 | 1.41E-238 |
| 64 | ***NEK7*** | 2.53E-41 | 0.525458 | 0.742 | 0.499 | 3.76E-37 | 1.48E-268 | 1.396506 | 0.974 | 0.799 | 2.21E-264 |
| 65 | ***GAD1*** | 6.95E-41 | 0.526193 | 0.999 | 0.987 | 1.03E-36 | 1.01E-191 | 0.786159 | 1 | 1 | 1.50E-187 |
| 66 | ***OSBPL1A*** | 7.78E-40 | 0.502789 | 0.992 | 0.976 | 1.16E-35 | 8.94E-112 | 0.519211 | 0.999 | 0.981 | 1.33E-107 |
| 67 | ***MAN1A2*** | 6.41E-39 | 0.588217 | 0.995 | 0.966 | 9.53E-35 | 1.02E-127 | 0.523014 | 0.994 | 0.951 | 1.52E-123 |
| 68 | ***GOT1*** | 9.75E-39 | 0.591727 | 0.89 | 0.696 | 1.45E-34 | 0 | 1.003105 | 1 | 1 | 0 |
| 69 | ***FAM81A*** | 2.96E-38 | 0.504891 | 0.929 | 0.831 | 4.40E-34 | 0 | 0.903627 | 0.994 | 0.5 | 0 |
| 70 | ***ZNF804B*** | 1.22E-37 | 1.373243 | 0.882 | 0.792 | 1.81E-33 | 1.16E-256 | 1.118871 | 0.869 | 0.425 | 1.73E-252 |
| 71 | ***AUH*** | 8.79E-37 | 0.527597 | 0.945 | 0.842 | 1.31E-32 | 3.95E-298 | 0.743486 | 0.999 | 0.99 | 5.88E-294 |
| 72 | ***ATP5B*** | 1.22E-33 | 0.645416 | 0.948 | 0.801 | 1.82E-29 | 4.50E-277 | 0.546393 | 1 | 1 | 6.68E-273 |

**2) List of conserved SST markers**

|  | genes | Human  _p_val | Human_avg  _log2FC | Human  _pct.1 | Human  _pct.2 | Human_p  _val_adj | Mouse  _p_val | Mouse_avg  _log2FC | Mouse  _pct.1 | Mouse  _pct.2 | Mouse_p  _val_adj |
| --- | --- | --- | --- | --- | --- | --- | --- | --- | --- | --- | --- |
| 1 | ***SYNPR*** | 3.36E-225 | -2.968149018 | 0.74 | 0.995 | 5.00E-221 | 0 | -3.67189 | 0.423 | 0.997 | 0 |
| 2 | ***GRID2*** | 2.72E-220 | -3.620336111 | 0.928 | 1 | 4.04E-216 | 2.88E-226 | -1.08124 | 0.888 | 0.983 | 4.29E-222 |
| 3 | ***SST*** | 9.40E-206 | -3.124805951 | 0.203 | 0.941 | 1.40E-201 | 0 | -5.24471 | 0.397 | 0.989 | 0 |
| 4 | ***CHRM3*** | 7.56E-201 | -2.50660055 | 0.959 | 1 | 1.12E-196 | 3.15E-149 | -1.04786 | 0.877 | 0.968 | 4.68E-145 |
| 5 | ***PDE1A*** | 1.36E-199 | -3.073489443 | 0.679 | 0.97 | 2.02E-195 | 0 | -2.24707 | 0.223 | 0.834 | 0 |
| 6 | ***RALYL*** | 3.82E-191 | -3.709768411 | 0.914 | 0.994 | 5.69E-187 | 4.70E-157 | -1.08611 | 0.928 | 0.988 | 6.99E-153 |
| 7 | ***CACNA2D3*** | 3.58E-179 | -2.978477201 | 0.886 | 0.99 | 5.32E-175 | 0 | -2.33148 | 0.423 | 0.971 | 0 |
| 8 | ***MAN1A1*** | 2.62E-156 | -1.926237376 | 0.277 | 0.836 | 3.90E-152 | 2.88E-136 | -0.77008 | 0.129 | 0.518 | 4.28E-132 |
| 9 | ***ELFN1*** | 1.16E-143 | -0.742491394 | 0.268 | 0.821 | 1.73E-139 | 0 | -0.90154 | 0.193 | 0.887 | 0 |
| 10 | ***NRXN3*** | 2.57E-143 | -0.803312232 | 1 | 1 | 3.82E-139 | 3.62E-82 | -0.64339 | 1 | 1 | 5.38E-78 |
| 11 | ***GRM1*** | 1.63E-130 | -2.18960719 | 0.554 | 0.896 | 2.43E-126 | 0 | -1.54176 | 0.379 | 0.944 | 0 |
| 12 | ***PRKCG*** | 4.31E-124 | -0.721701666 | 0.398 | 0.851 | 6.40E-120 | 0 | -1.34398 | 0.451 | 0.981 | 0 |
| 13 | ***TRHDE*** | 4.40E-120 | -2.705044005 | 0.732 | 0.939 | 6.55E-116 | 0 | -1.96921 | 0.533 | 0.963 | 0 |
| 14 | ***LHFPL3*** | 1.06E-118 | -1.944637698 | 0.847 | 0.964 | 1.57E-114 | 1.68E-121 | -0.63719 | 0.503 | 0.775 | 2.50E-117 |
| 15 | ***GRIN3A*** | 1.53E-112 | -2.05172379 | 0.651 | 0.932 | 2.28E-108 | 0 | -2.25637 | 0.293 | 0.984 | 0 |
| 16 | ***SEMA6A*** | 2.13E-110 | -1.193836197 | 0.309 | 0.8 | 3.17E-106 | 8.98E-119 | -0.50772 | 0.577 | 0.825 | 1.34E-114 |
| 17 | ***RGS6*** | 1.24E-106 | -2.515207405 | 0.73 | 0.895 | 1.85E-102 | 3.29E-287 | -1.13604 | 0.272 | 0.809 | 4.89E-283 |
| 18 | ***MGAT4C*** | 6.63E-101 | -1.730349102 | 0.963 | 0.997 | 9.87E-97 | 3.57E-118 | -1.59739 | 0.955 | 0.945 | 5.31E-114 |
| 19 | ***SHISA6*** | 8.81E-97 | -1.472758926 | 0.806 | 0.967 | 1.31E-92 | 2.96E-256 | -0.5483 | 0.103 | 0.692 | 4.40E-252 |
| 20 | ***CACNA1E*** | 1.41E-93 | -1.124521716 | 0.522 | 0.885 | 2.09E-89 | 4.70E-208 | -0.8622 | 0.42 | 0.869 | 6.99E-204 |
| 21 | ***SLC24A3*** | 9.85E-93 | -1.562706852 | 0.746 | 0.927 | 1.47E-88 | 9.52E-165 | -0.97419 | 0.8 | 0.963 | 1.42E-160 |
| 22 | ***RUNX1T1*** | 3.11E-91 | -1.139766315 | 0.957 | 0.996 | 4.63E-87 | 5.89E-230 | -0.78085 | 0.944 | 0.997 | 8.76E-226 |
| 23 | ***FSTL5*** | 4.11E-89 | -1.724521009 | 0.876 | 0.967 | 6.11E-85 | 9.76E-199 | -0.83386 | 0.609 | 0.925 | 1.45E-194 |
| 24 | ***LRRC7*** | 6.55E-88 | -1.066506985 | 0.995 | 1 | 9.74E-84 | 9.71E-139 | -0.74866 | 0.957 | 0.992 | 1.44E-134 |
| 25 | ***STXBP6*** | 2.12E-83 | -1.126453338 | 0.874 | 0.986 | 3.16E-79 | 3.72E-141 | -0.7105 | 0.628 | 0.974 | 5.54E-137 |
| 26 | ***NETO1*** | 3.08E-82 | -1.222075475 | 0.897 | 0.98 | 4.58E-78 | 8.42E-308 | -1.15495 | 0.938 | 0.997 | 1.25E-303 |
| 27 | ***FXYD6*** | 4.75E-82 | -1.18047688 | 0.612 | 0.924 | 7.06E-78 | 1.56E-160 | -1.69249 | 0.294 | 0.733 | 2.32E-156 |
| 28 | ***ILDR2*** | 1.38E-81 | -0.593372424 | 0.079 | 0.519 | 2.05E-77 | 2.24E-296 | -0.7458 | 0.391 | 0.942 | 3.33E-292 |
| 29 | ***TMEFF2*** | 5.01E-81 | -1.325462733 | 0.742 | 0.944 | 7.45E-77 | 4.21E-179 | -1.3862 | 0.904 | 0.969 | 6.26E-175 |
| 30 | ***UNC13C*** | 3.70E-79 | -1.673687351 | 0.711 | 0.889 | 5.50E-75 | 1.87E-210 | -1.27887 | 0.161 | 0.657 | 2.78E-206 |
| 31 | ***CACNG3*** | 1.07E-78 | -1.086206439 | 0.798 | 0.962 | 1.58E-74 | 5.05E-228 | -1.08848 | 0.862 | 0.957 | 7.50E-224 |
| 32 | ***NELL1*** | 1.68E-78 | -2.529424053 | 0.715 | 0.89 | 2.50E-74 | 3.33E-21 | -0.52478 | 0.63 | 0.691 | 4.95E-17 |
| 33 | ***GNG2*** | 5.29E-77 | -0.865491881 | 0.4 | 0.804 | 7.87E-73 | 4.54E-281 | -0.8967 | 0.951 | 0.999 | 6.74E-277 |
| 34 | ***GPM6A*** | 2.75E-75 | -0.98530689 | 0.999 | 1 | 4.08E-71 | 3.04E-281 | -0.9099 | 1 | 1 | 4.52E-277 |
| 35 | ***PCDH11X*** | 1.36E-68 | -1.324603093 | 0.912 | 0.989 | 2.02E-64 | 2.10E-103 | -0.84013 | 0.633 | 0.859 | 3.12E-99 |
| 36 | ***GDA*** | 6.31E-68 | -0.900191083 | 0.549 | 0.777 | 9.38E-64 | 0 | -0.95308 | 0.151 | 0.881 | 0 |
| 37 | ***SCN3B*** | 4.15E-65 | -0.788329076 | 0.495 | 0.865 | 6.18E-61 | 2.29E-243 | -0.69824 | 0.42 | 0.909 | 3.40E-239 |
| 38 | ***GALNT14*** | 7.74E-63 | -1.266147678 | 0.605 | 0.862 | 1.15E-58 | 0 | -1.14271 | 0.154 | 0.851 | 0 |
| 39 | ***RELN*** | 2.81E-61 | -1.809691022 | 0.755 | 0.889 | 4.18E-57 | 2.94E-276 | -2.22861 | 0.372 | 0.846 | 4.37E-272 |
| 40 | ***TRPC6*** | 6.73E-61 | -1.208845839 | 0.297 | 0.635 | 1.00E-56 | 7.95E-192 | -0.81462 | 0.132 | 0.623 | 1.18E-187 |
| 41 | ***GAP43*** | 7.07E-61 | -0.924833979 | 0.747 | 0.937 | 1.05E-56 | 6.72E-291 | -1.44211 | 0.794 | 0.982 | 9.99E-287 |
| 42 | ***BCL11A*** | 2.10E-57 | -0.992104217 | 0.81 | 0.96 | 3.13E-53 | 2.27E-231 | -0.908 | 0.794 | 0.972 | 3.38E-227 |
| 43 | ***NETO2*** | 3.81E-57 | -1.101550987 | 0.916 | 0.961 | 5.66E-53 | 6.40E-66 | -0.5155 | 0.867 | 0.97 | 9.51E-62 |
| 44 | ***GNG4*** | 3.59E-56 | -0.543420253 | 0.588 | 0.822 | 5.34E-52 | 1.64E-126 | -0.6928 | 0.559 | 0.847 | 2.43E-122 |
| 45 | ***NPAS3*** | 5.95E-56 | -1.751672833 | 0.996 | 0.992 | 8.85E-52 | 5.56E-129 | -1.09354 | 0.824 | 0.948 | 8.26E-125 |
| 46 | ***CPLX2*** | 2.71E-53 | -0.526370408 | 0.438 | 0.745 | 4.03E-49 | 0 | -1.00282 | 0.435 | 0.968 | 0 |
| 47 | ***CDH4*** | 7.10E-50 | -0.919760174 | 0.775 | 0.925 | 1.06E-45 | 1.62E-171 | -0.51596 | 0.3 | 0.79 | 2.41E-167 |
| 48 | ***RPH3A*** | 6.34E-47 | -0.842437813 | 0.782 | 0.926 | 9.43E-43 | 1.72E-225 | -0.66075 | 0.744 | 0.982 | 2.56E-221 |
| 49 | ***LINGO2*** | 4.06E-46 | -2.157020287 | 0.933 | 0.969 | 6.03E-42 | 4.37E-127 | -1.19095 | 0.515 | 0.767 | 6.50E-123 |
| 50 | ***OPRM1*** | 3.64E-45 | -1.021805121 | 0.361 | 0.622 | 5.41E-41 | 3.60E-199 | -0.80843 | 0.33 | 0.826 | 5.36E-195 |
| 51 | ***DAPK1*** | 2.67E-43 | -0.781840914 | 0.965 | 0.995 | 3.97E-39 | 3.30E-159 | -0.57581 | 0.873 | 0.985 | 4.90E-155 |
| 52 | ***CDH13*** | 1.94E-38 | -1.155828791 | 1 | 1 | 2.88E-34 | 2.28E-107 | -1.28473 | 0.886 | 0.921 | 3.38E-103 |
| 53 | ***CBLN4*** | 5.98E-38 | -0.890734091 | 0.111 | 0.378 | 8.89E-34 | 7.17E-57 | -0.75032 | 0.084 | 0.302 | 1.07E-52 |
| 54 | ***GAS7*** | 7.06E-36 | -0.616038151 | 0.906 | 0.949 | 1.05E-31 | 2.52E-138 | -0.60424 | 0.978 | 0.99 | 3.74E-134 |
| 55 | ***CDH9*** | 2.41E-35 | -0.886093138 | 0.949 | 0.994 | 3.58E-31 | 4.19E-54 | -1.20642 | 0.695 | 0.731 | 6.24E-50 |
| 56 | ***AGPAT4*** | 1.07E-31 | -0.7173287 | 0.854 | 0.947 | 1.60E-27 | 1.15E-206 | -0.61242 | 0.57 | 0.947 | 1.70E-202 |
| 57 | ***PENK*** | 1.39E-31 | -0.622883675 | 0.013 | 0.199 | 2.07E-27 | 3.79E-24 | -0.90162 | 0.246 | 0.399 | 5.64E-20 |
| 58 | ***NCAM2*** | 7.40E-31 | -1.781616538 | 0.881 | 0.936 | 1.10E-26 | 1.43E-31 | -0.65842 | 0.61 | 0.717 | 2.13E-27 |
| 59 | ***GRIK3*** | 2.04E-30 | -0.61276681 | 0.963 | 0.985 | 3.04E-26 | 2.54E-256 | -0.7638 | 0.856 | 0.98 | 3.77E-252 |
| 60 | ***ADCY2*** | 1.77E-28 | -0.702720849 | 0.976 | 0.977 | 2.63E-24 | 0 | -1.96678 | 0.45 | 0.896 | 0 |
| 61 | ***SPON1*** | 2.48E-28 | -0.801761274 | 0.558 | 0.705 | 3.69E-24 | 3.14E-132 | -1.22058 | 0.31 | 0.668 | 4.66E-128 |
| 62 | ***NKAIN3*** | 5.49E-28 | -1.294489881 | 0.76 | 0.864 | 8.17E-24 | 3.03E-49 | -0.8815 | 0.32 | 0.51 | 4.51E-45 |
| 63 | ***BASP1*** | 1.65E-27 | -0.536657805 | 0.739 | 0.88 | 2.45E-23 | 1.21E-305 | -0.91297 | 0.999 | 1 | 1.80E-301 |
| 64 | ***DGKG*** | 2.36E-26 | -1.139533565 | 0.649 | 0.778 | 3.51E-22 | 2.62E-264 | -1.04 | 0.893 | 0.997 | 3.89E-260 |
| 65 | ***NPY*** | 2.31E-25 | -0.747647207 | 0.036 | 0.209 | 3.43E-21 | 1.24E-93 | -2.85621 | 0.692 | 0.877 | 1.84E-89 |
| 66 | ***RAB3B*** | 1.02E-24 | -0.570845259 | 0.672 | 0.837 | 1.51E-20 | 0 | -1.08174 | 1 | 1 | 0 |
| 67 | ***TSPAN7*** | 9.37E-21 | -0.716187962 | 0.993 | 0.987 | 1.39E-16 | 0 | -0.70471 | 1 | 1 | 0 |
| 68 | ***PTPRR*** | 7.10E-20 | -0.701439849 | 0.871 | 0.92 | 1.06E-15 | 1.98E-82 | -0.50409 | 0.731 | 0.899 | 2.94E-78 |
| 69 | ***NAV2*** | 1.06E-19 | -0.529547081 | 0.999 | 1 | 1.57E-15 | 2.80E-46 | -0.51168 | 0.975 | 0.989 | 4.17E-42 |
| 70 | ***CHL1*** | 7.12E-17 | -0.611171387 | 0.881 | 0.916 | 1.06E-12 | 1.12E-256 | -1.15371 | 0.971 | 0.997 | 1.66E-252 |
| 71 | ***KCND3*** | 1.72E-16 | -0.566983771 | 0.736 | 0.821 | 2.55E-12 | 1.04E-248 | -0.7154 | 0.433 | 0.898 | 1.55E-244 |
| 72 | ***RGS17*** | 4.54E-12 | -0.564465103 | 0.644 | 0.728 | 6.75E-08 | 0 | -1.20991 | 0.943 | 0.997 | 0 |
| 73 | ***PGRMC1*** | 5.81E-11 | -0.529196586 | 0.509 | 0.622 | 8.64E-07 | 3.29E-280 | -0.81598 | 0.999 | 1 | 4.89E-276 |
| 74 | ***DYNLL1*** | 8.23E-11 | -0.519853887 | 0.77 | 0.847 | 1.22E-06 | 0 | -1.03944 | 1 | 1 | 0 |
| 75 | ***SEMA3E*** | 7.55E-10 | -0.549336522 | 0.375 | 0.476 | 1.12E-05 | 2.05E-68 | -0.56947 | 0.491 | 0.721 | 3.05E-64 |
