# Supplementary material for: Target cell-specific synaptic dynamics of excitatory to inhibitory neuron connections in supragranular layers of human neocortex
Source: eLife. 2023 May 30;12:e81863. doi: 10.7554/eLife.81863 (PMC10332811; doi:10.7554/eLife.81863)
Supplement: Supplementary file 2. [file elife-81863-supp2.docx]

**Supplementary File 2. List of intrinsic membrane properties classifier features and linear discriminant analysis (LDA) coefficients.**

| **Feature name** | **LDA coefficient** |
| --- | --- |
| AP height (rheobase) | -1.018102 |
| depolarizing sag ratio | 0.94688 |
| AP upstroke adaptation ratio | 0.696121 |
| membrane time constant | -0.586977 |
| f-I curve slope | 0.446189 |
| AP downstroke adaptation ratio | 0.393441 |
| input resistance (transient) | -0.381634 |
| AP threshold adaptation ratio | -0.376394 |
| AHP voltage (rheobase) | -0.298523 |
| ISI CV (mean) | 0.284843 |
| AHP voltage adaptation ratio | 0.219969 |
| AP width adaptation ratio | -0.197607 |
| input resistance (steady-state) | -0.192592 |
| AP height adaptation ratio | -0.153465 |
| adaptation index (mean) | -0.146462 |
| depolarizing sag peak time | -0.138321 |
| mean ISI (rheo+40 pA) | 0.11141 |
| ISI adaptation ratio | 0.110781 |
| adaptation index (rheo+40 pA) | -0.083049 |
| ISI CV (rheo+40 pA) | -0.079883 |
| first AP latency (rheobase) | 0.059851 |
| sag peak time | 0.055374 |
| AP height (rheo+40 pA) | -0.027617 |
| first AP latency (rheo+40 pA) | -0.018279 |
